# Understanding fluorescence time curves during ileal pouch-anal anastomosis with or without vascular ligation

**DOI:** 10.1007/s00464-023-09921-y

**Published:** 2023-03-14

**Authors:** J. J. Joosten, M. D. Slooter, R. M. van den Elzen, P. R. Bloemen, W. Laméris, D. M. de Bruin, W. A. Bemelman, R. Hompes

**Affiliations:** 1 Department of Surgery, Amsterdam University Medical Centres (UMC), Academic Medical Centre (AMC), Postbox 22660, 1100 DD Amsterdam, The Netherlands; 2 Cancer Center Amsterdam, Imaging and Biomarkers, Amsterdam, The Netherlands; 3 Department of Radiology, Amsterdam UMC Location University of Amsterdam, Amsterdam, The Netherlands; 4 Department of Biomedical Engineering, Amsterdam UMC Location University of Amsterdam, Amsterdam, The Netherlands; 5 Department of Surgery, Amsterdam UMC Location Vrije Universiteit Amsterdam, Amsterdam, The Netherlands

**Keywords:** Fluorescence angiography (FA), Fluorescence time curves, Indocyanine green (ICG), Ileal pouch-anal anastomosis (IPAA), Vascular ligation, Anastomotic leakage

## Abstract

**Background:**

Intraoperative indocyanine green fluorescence angiography (ICG-FA) may be of added value during pouch surgery, in particular after vascular ligations as lengthening maneuver. The aim was to determine quantitative perfusion parameters within the efferent/afferent loop and explore the impact of vascular ligation. Perfusion parameters were also compared in patients with and without anastomotic leakage (AL).

**Methods:**

All consenting patients that underwent FA-guided ileal pouch-anal anastomosis (IPAA) between July 2020 and December 2021 were included. After intravenous bolus injection of 0.1 mg/kg ICG, the near-infrared camera (Stryker Aim 1688) registered the fluorescence intensity over time. Quantitative analysis of ICG-FA from standardized regions of interests on the pouch was performed using software. Fluorescence parameters were extracted for inflow (*T*_0_, *T*_max_, *F*_max_, slope, Time-to-peak) and outflow (*T*_90%_ and *T*_80%_). Change of management related to FA findings and AL rates were recorded.

**Results:**

Twenty-one patients were included, three patients (14%) required vascular ligation to obtain additional length, by ligating terminal ileal branches in two and the ileocolic artery (ICA) in one patient. In nine patients the ICA was already ligated during subtotal colectomy. ICG-FA triggered a change of management in 19% of patients (*n* = 4/21), all of them had impaired vascular supply (ligated ileocolic/ terminal ileal branches). Overall, patients with intact vascular supply had similar perfusion patterns for the afferent and efferent loop. Pouches with ICA ligation had longer *T*_max_ in both afferent as efferent loop than pouches with intact ICA (afferent 51 and efferent 53 versus 41 and 43 s respectively). Mean slope of the efferent loop diminished in ICA ligated patients 1.5(IQR 0.8–4.4) versus 2.2 (1.3–3.6) in ICA intact patients.

**Conclusion:**

Quantitative analysis of ICG-FA perfusion during IPAA is feasible and reflects the ligation of the supplying vessels.

**Supplementary Information:**

The online version contains supplementary material available at 10.1007/s00464-023-09921-y.

Ileal pouch-anal anastomosis (IPAA) helps to restore continuity after proctocolectomy for patients with ulcerative colitis, familial adenomatous polyposis and well selected patients with Crohn’s colitis [1]. After pouch surgery, a feared complication is anastomotic leakage (AL) which can occur in up to 15% of patients [2, 3]. A traction-free, well-vascularized anastomosis is essential for anastomotic healing, and in pouch surgery these two factors need to be carefully balanced. In order to have a tension free anastomosis, lengthening maneuvers may require vascular ligation [4] of the ileocolic artery(ICA) or ileal arterial branches. In most patients the ICA is already compromised at initial subtotal colectomy, and further vascular ligations may even have a bigger impact.

Intraoperative fluorescence angiography using indocyanine green (ICG-FA) is widely applied to assess tissue perfusion and could contribute to the prevention of AL secondary to perfusion restriction [5, 6]. During IPAA, ICG-FA may be of added value as it can guide safe vascular ligations without increasing the incidence of AL [7, 8]. However, the interpretation of ICG-FA remains mostly subjective, and quantification of ICG-FA remains a challenge [9, 10]. First efforts in quantifying the fluorescence signal during IPAA surgery support that time from ICG administration to fluorescent enhancement of the afferent and efferent loop may be prolonged in patients with vascular ligation [7]. Outflow on the other hand may also provide valuable information, as outflow problems are correlated to venous congestion contributing to ischemia [11]. Taking this into account, it is important to focus on both inflow as outflow parameters.

The objective of the present pilot study is to determine quantitative fluorescent parameters to assess in- and out-flow in relation to vascular ligation during IPAA surgery. Intraoperative change of management related to ICG-FA and AL were recorded.

## Materials and methods

### Study design

This case series has been reported in line with the PROCESS Guideline [12]. In this single center pilot study, we included all consenting patients that underwent FA-guided IPAA in Amsterdam UMC from July 2020 until December 2021.

Patients were included when they met the following criteria: 18 years or older, proctocolectomy or completion proctectomy with (redo) IPAA for inflammatory bowel disease or inherited colorectal cancer disorders. Patients were excluded in case of allergy to ICG, iodide or sodium iodide. ICG-FA data were recorded in a prospectively maintained database, along with patient data from the electronic patient system.

The Institutional Review Board of the Amsterdam University Medical Centres (UMC), location Academic Medical Centre (AMC), approved the study protocol and confirmed that the Medical Research lnvolving Human Subjects Act (WMO) did not apply.

### Surgical procedure

Different strategies for restorative proctocolectomy with IPAA were applied, including 1 and modified-2-stage procedures [13]. The modified 2-stage procedure was the standard approach after prior subtotal colectomy for refractory disease. In patients who had their subtotal colectomy at Amsterdam Medical Centres, the ileocolic arcade was preserved as a routine. In patients referred from other units, the ileocolic arcade was mostly not preserved, and this was verified through the operative notes and postoperative abdominal CT if available.

**Fig. 1:**
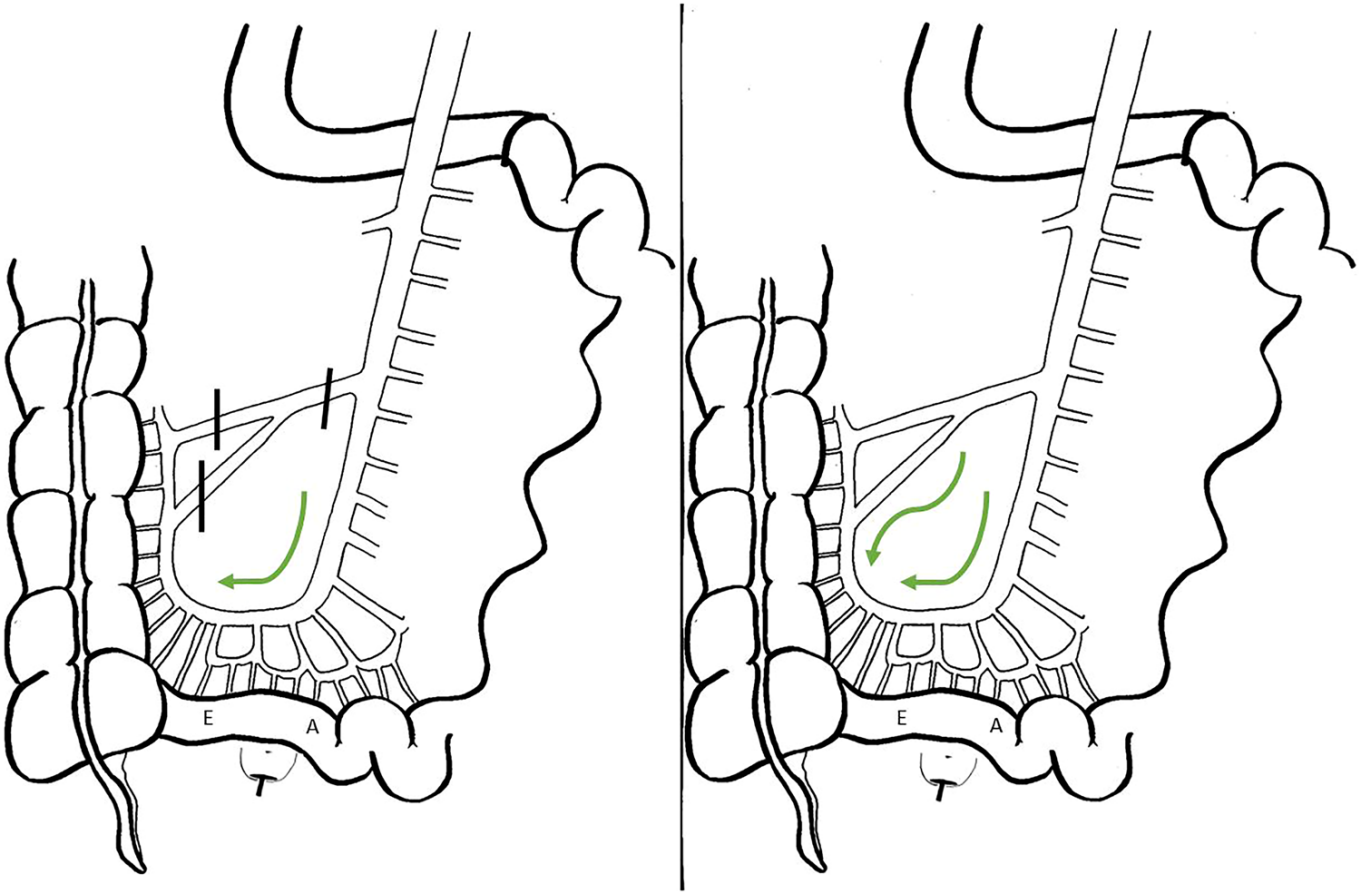
Route of ICG in case of ligation of the ileocolic artery (left) and an intact ileocolic artery (right) with E representing the efferent and A the afferent loop of the pouch. Adapted with permission from Slooter et al. (7)

Completion proctectomy and IPAA were performed by a combined abdominal and transanal minimally invasive approach as described previously [11]. Standard lengthening maneuvers consisted of dividing all adhesions, mobilization of the terminal ileal mesentery to the level of the duodenum and transverse peritoneal incisions on both sides of the small bowel mesentery. The yardstick for sufficient length was that the apex of the pouch should reach 1–2 cm below the pubic bone. If length was considered insufficient, the ICA and/or interconnecting terminal ileal branches were ligated (Fig. 1). After securing sufficient length, the J-pouch was constructed by a side-to-side ileal anastomosis of 10 cm length using a linear stapler. A double purse string single staple anastomosis was performed as a routine with suture reinforcement. A diverting ileostomy was only created in case of technical problems (e.g., positive reverse leak test or subjective feeling of tension on the anastomosis) or aberrant ICG-FA findings (i.e., delayed fluorescence enhancement based on subjective interpretation of the surgeon).

## Standardized fluorescent assessment

Perfusion of the pouch was assessed extracorporeally before connection of anvil and stapler for the anastomosis. The operating table was placed in a neutral position, the laparoscopic Stryker 1688 camera system (Stryker, Kalamazoo, MI, U.S.A.), 30 degree optic was placed in a fixed position 9 cm perpendicular from the anvil in the top of the pouch. All light in the operation room was switched off to minimize external light reflection. ICG (Verdye, Diagnostic Green; 0.1 mg/kg/bolus) was injected peripherally as a bolus directly in the iv catheter of the left arm without iv extension. Starting with the moment of ICG injection, perfusion was captured in a continuous video recording for 200 s.

### Quantification of fluorescent imaging

**Fig. 2:**
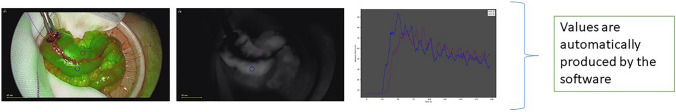
J-pouch and example how the ROI selection is made and curve generated

**Fig. 3:**
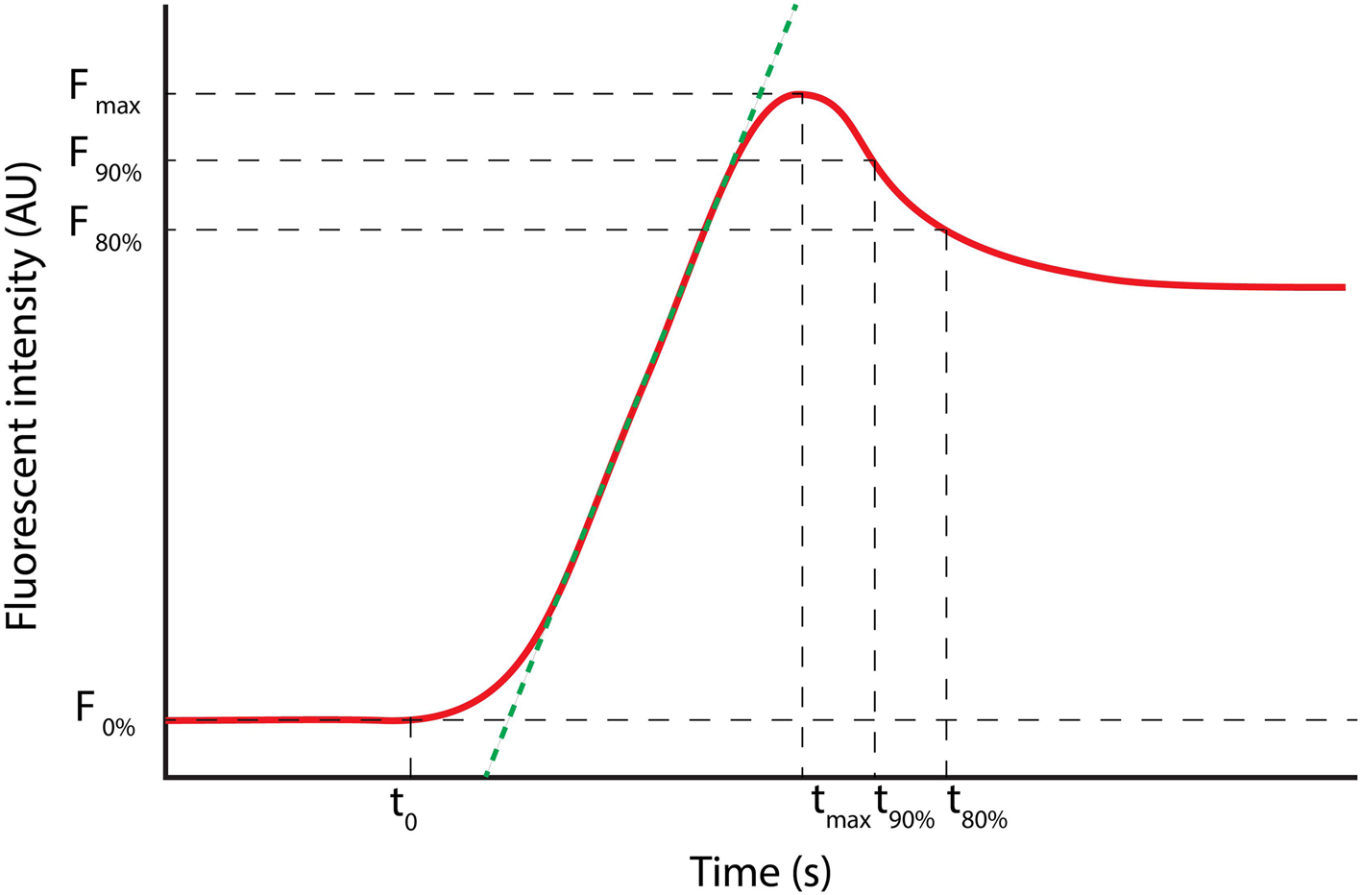
Fluorescent parameters: Influx time point (*t*_0_): the time point at which the fluorescence intensity in the ROI was statistically significantly larger than the background, *F*_max_: maximal intensity in arbitrary units (AU), *t*_max_: time in at which the background-corrected fluorescence intensity reached *F*_max_, time-to-peak (ttp): *T*_max_–*t*_0_, the green line represents the mean slope: rate at which the fluorescence intensity increased (AU/s), t_90%_: time in seconds after *F*_max_ until 90% of *F*_max_ has been reached, t_80%_: time in seconds after *F*_max_ until 80% of *F*_max_ has been reached

In order to achieve objective quantification, the raw ICG-FA video data were analyzed post hoc by tailor made software written in Python (COPYRIGHT)**.** After loading the video into the software, size was calibrated using a measuring tape which was placed in the frame. Subsequently, a circular region of interests (ROI) with a diameter of 1 cm was placed in the middle of the pouch body, both on the afferent and efferent loop (Fig. 2). The software subsequently extracted the mean intensity within the ROI for every frame and plotted the ICG in- and out-flow in a fluorescence–time curve. A slightly modified version of the arterial input function reported by Elliott et al. was fitted to the curve to reduce the influence of noise on the calculated parameters [14]. From this fit, the following parameters were extracted (Fig. 3): Influx time point (*t*_0_): the time point at which the fit started, thereby increasing to above baseline intensity, *F*_max_: maximal intensity in arbitrary units (AU), *T*_max_: time in seconds from ICG administration until *F*_max_ has been reached., time-to-peak (ttp); time in seconds from τo until *F*_max_ has been reached, mean slope: mean rate at which the fluorescence intensity increased between *t*_0_ and *T*_max_ (AU/s), *T*_90%_: time in seconds after *F*_max_ until fluorescence intensity has dropped to 90% of *F*_max_ and *T*_80%_: time in seconds after *F*_max_ until fluorescence intensity has dropped to 80% of *F*_max_.

### Outcomes

The primary outcome was to determine quantitative perfusion parameters during IPAA within the efferent/afferent loop and explore the impact of lengthening measures requiring vascular ligation or previous inadvertent interruption of the ileocolic arcade. Perfusion parameters in patients with AL were also compared to those without.

Secondary outcomes included change in management due to FA, AL within 90 days and reinterventions due to AL. Change in management by FA was defined as every measure taken based on the results of FA only, i.e., suture reinforcement of hypo-perfused regions, additional resections or preserving the pouch and creating an ileostomy. Interpretation of FA was mainly based on presence and absence of fluorescent signal in parts of the pouch. AL was recorded when an anastomotic defect was objectified by CT-scan or during endoscopy and was graded according to impact on clinical management similar to Sahami et al. [3].

### Statistics

Patient baseline characteristics and imaging characteristics are summarized using simple descriptive statistics. All categorical data will be presented as number of cases and percentages, while continuous data will be shown as either mean ± standard deviation or as median and interquartile range (IQR), depending on the data distribution. Data was analyzed using the Statistical Package for Social Sciences (SPSS) of IBM Statistics, version 26.0, or the latest version.

## Results

### Baseline characteristics

In total, 21 patients were included in this study with a mean age of 40.5 ± 11.3 years at time of IPAA. Half of the patients were male (52.4%). Patient characteristics are outlined in Table 1.

**Table 1:** Baseline characteristics

	IPAA (*n* = 21)	AL (*n* = 3)	No AL (*n* = 18)
Gender, male	11 (52.4)	1/3	10/18
Age (years) mean ± SD	40.5 ± 11.3	37.3 ± 11.0	41.0 ± 11.6
BMI (kg/m^2^) mean ± SD	25.8 + 4.9	25.4 ± 4.1	25.9 ± 5.1
ASA classification (≤ 2)	18 (85.7)	2/3	16/18
Smoker (active)	1 (4.8)	0	1/18
Comorbidity*			
Vascular	0	0	0
Diabetes Mellitus	2 (9.5)	0	2/18
Diagnosis			
Ulcerative Colitis #	19 (90.5)	3/3	16/18
FAP	2 (9.5)	0	2/18
Medication for UC			
None	16/19	3/3	13/16
Mesalazine	2/19	0	2/16
Biologicals	1/19	0	1/16

Data is shown in *n* (%), unless otherwise stated, *ASA* BMI body-mass index, *IPAA* ileal pouch-anal anastomosis

^*^Vascular comorbidity: brain infraction, myocardial infarction, or peripheral vascular disease. Diabetes Mellitus: type 1 or 2. # of whom one had secondary sigmoid carcinoma and one multifocal dysplasia, *FAP* Familial adenomatous polyposis, *UC* ulcerative Colitis

### Operative characteristics

Operative characteristics are outlined in Table 2. The abdominal approach was in all except two patients via laparoscopy, requiring conversion into a laparotomy in one patient due to the extensiveness of dense adhesions. All patients underwent transanal minimally invasive surgery for the proctectomy, rectal cuff mobilization, and double purse string single stapled anastomosis.

**Table 2:** Operative characteristics

	FA-guided IPAA (*n* = 21)
Prior surgery definite ileocolic vessel ligation	9 (42.9)
Stage procedure	
1-Stage	1 (4.8)
2-Stage	1 (4.8)
Modified 2-stage	14 (66.7)
3-Stage	4 (23.8)
Other*	1 (4.8)
Abdominal approach	
Laparoscopy	19 (90.5)
Open	2 (9.5)
Transanal approach	
TAMIS	21 (100)
Abdominal conversion	1 (4.8)
Mobilization	
Mesenteric incisions	21 (100)
Intraoperative vessel ligation	3 (9.6)
Ileocolic vessel ligation	1 (4.8)
Interconnecting terminal branches	2 (4.8)
Construction anastomosis	
Hand-sewn	0
Stapled	21 (100)
Size stapler (mm)	
28	0
29	4 (19)
31	1 (4.8)
32	16 (76.2)
Median distance to DL in cm (IQR)	2 (1–2)
Operative time	240 (230–213)
Intraoperative complications*	0

Data is shown in *n* (%) or *n*/*n*), unless otherwise stated, *redo pouch due to FAP overgrowth in the initial pouch

*TAMIS* transanal minimally invasive surgery**,** *DL* dentate line

Intraoperative complications included: bleeding > 0.5L, iatrogenic uretral injury, serosal laesion, bowel perforation

### Vascular ligation

In almost half of the patients the ICA had already been ligated during the previous subtotal colectomy. During IPAA, intraoperative vessel ligation was performed in three patients to obtain sufficient reach for anastomosis, consisting of ligation of the ICA in one patient and interconnecting terminal ileal branches in two patients. Both patients with ligation of interconnecting terminal ileal branches had intact ICA.

### Fluorescence outcomes

#### Vascular ligation group

**Fig. 4:**
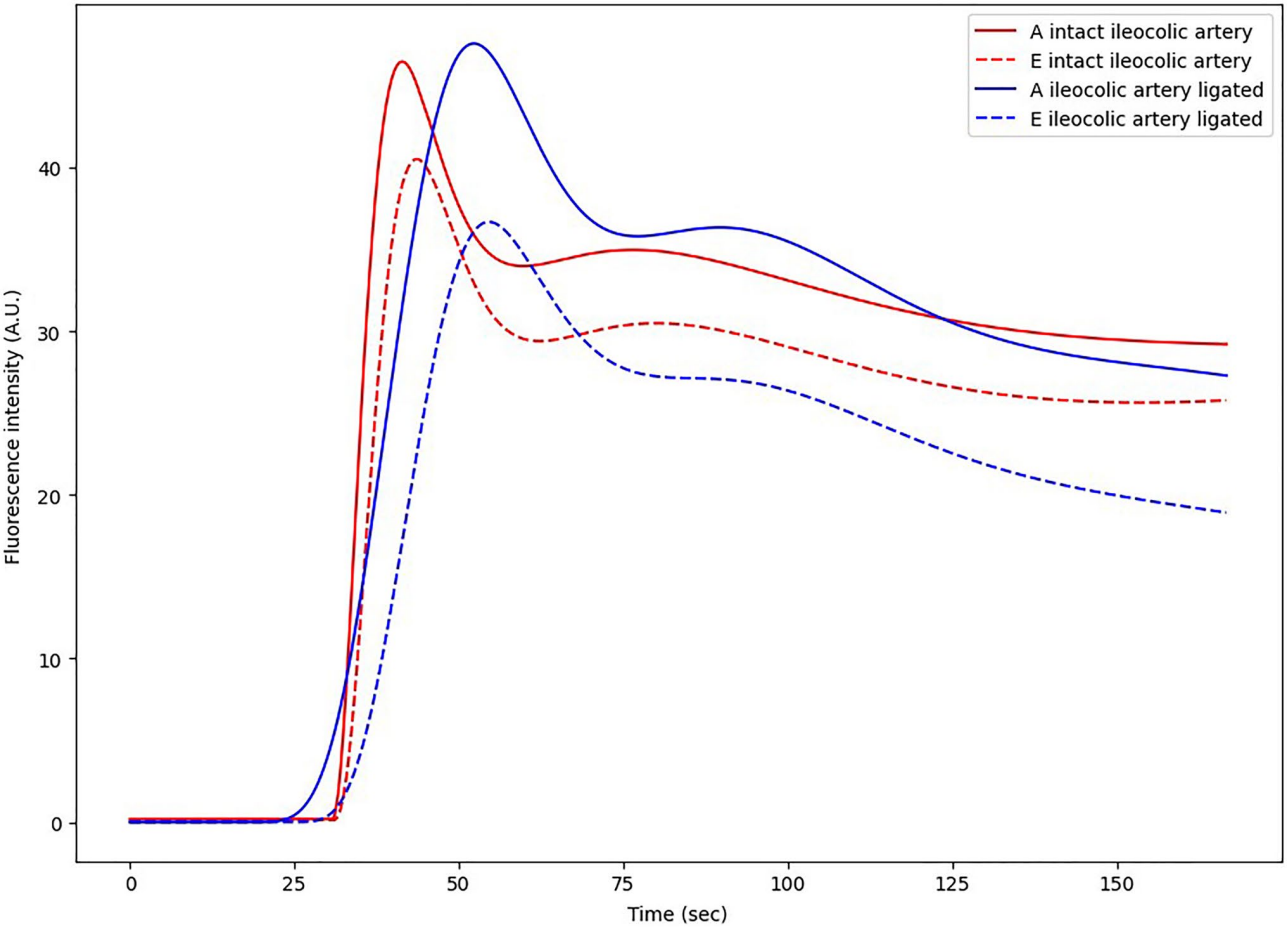
Curves based on the median value of fluorescence parameters of the group with and without ligation of the ileocolic arcade in the afferent and efferent loop

In Table 3 the fluorescent parameters are summarized for patients with an intact or interrupted ICA. In patients without vascular ligation time values were similar for the afferent and efferent small bowel loops. When comparing the ICA ligated to intact ICA pouches, the time to achieve maximum fluorescent intensity (*T*_max_) was 10 s longer in both the afferent as efferent loop of the pouch (afferent 51 and efferent 53 versus 41 and 43 s respectively). In case of ICA ligation the mean slope of the efferent loop was less steep 1.5 AU/s (IQR 0.8–4.4) versus 2.2 AU/s (IQR 1.3–3.6) without ligation. Regarding the outflow parameters: longer time intervals were observed to reach 80% of *F*_max_ in the ligation group (10 versus 16 s respectively). In Fig. 4 curves based on the median values among the two groups are represented, despite a difference in fluorescence intensity the curves of the afferent and efferent loops of the two groups have similar shapes during in- and out-flow.

**Table 3:** Fluorescent parameters for patients with an intact ileocolic arcade and the ileocolic artery ligated during prior surgery or intraoperatively

Fluorescence parameter	ICA intact (*n* = 11)	ICA ligated (*n* = 10)
Afferent loop		
Inflow parameters		
* t*_0_ (s)	29 (23–35)	27 (16–51)
* t*_max_ (s)	41 (36–58)	51 (31–71)
* t*_tp_ (s)	17 (12–24)	17 (8–36)
* F*_max_ (AU)	46 (39–57)	49 (33–97)
Slope (AU/s)	2.4 (2.2–3.5)	2.1 (1.4–3.1)
Outflow parameters		
* t*_90%_ (s)	5 (5–10)	6 (5–10)
* t*_80%_ (s)	10 (8–20)	14 (9–20)
Efferent loop		
Inflow parameters		
* t*_0_ (s)	30 (24–35)	31 (24–71)
* t*_max_ (s)	43 (36–65)	53 (34–64)
ttp (s)	20 (9–24)	17 (10–28)
* F*_max_ (AU)	40 (32–71)	37 (25–77)
Slope (AU/s)	2.2 (1.3–3.6)	1.5 (0.8–4.4)
Outflow parameters		
* t*_90%_ (s)	6 (5–11)	8 (5–17)
* t*_80%_ (s)	10 (7–18)	16 (9–31)

*ICA* ileocolic artery, *ttp* time-to-peak, *AU* arbitrary units, *slope* mean slope

Both patients in whom the interconnecting terminal ileal branches were ligated had macroscopic blue colorization of the efferent loop during the surgery. In one patient, *T*_max_ in both the afferent as efferent loop was prolonged (see Supplementary Table 1) with *T*_max_ of 67 s for efferent loop, but the bowel loops showed clear fluorescence enhancement (*F*_max_ 84 for afferent and 71 for efferent loop). In both patients, outflow was prolonged with T_80%_ in the afferent loop of 13 and in the efferent loop of 36 s for one patient and. 23 and 71 s for the other.

#### Change of management due to ICG-FA assessment

Based on qualitative assessment of the ICG-FA, change of management was opted in four out of 21 patients (19%). Of these, two patients had prior ICA ligation and two patients necessitated intraoperative ligation of the interconnecting terminal ileal branches to obtain more length. One patient with prior ICA ligation had delayed fluorescent enhancement of the entire pouch, with no fluorescent enhancement in the apex of the pouch. This region was inverted by sutures. In retrospect, flat slopes in both the afferent as efferent loop (0.75 and 0.64) were observed. This patient developed an AL on postoperative day 16. In the second patient with prior ICA ligation, there was a delayed fluorescent enhancement of the efferent loop of the pouch. The anastomosis was constructed, and a protective ileostomy was created, switching from a planned modified 2-stage into a 3-stage procedure. In one patient after ligation of the interconnecting terminal branches, a delayed enhancement during ICG-FA was observed in the apex of the pouch, which was not observed by white light assessment, therefore this hypoperfused area was inverted by sutures. In the other patient after ligation of the interconnecting terminal ileal branches, the pouch body discolored gradually in white light, but subsequent ICG-FA showed uniform and rapid enhancement. The operative plan was changes from a 1-stage into a 2-stage procedure. The latter three patients had an uncomplicated postoperative course.

#### Postoperative outcomes

Fluorescence parameters are summarized in Table 4, in which the results are also shown for patients with or without AL separately. AL was observed in three out of 21 patients (14%), all located at the circular anastomosis and diagnosed on postoperative day 5, 12, and 16. All patients did not have an ileostomy at moment of diagnosis. Two of the AL were diagnosed by endoscopic examination and the remaining one by CT scan. All patients were treated by creating an ileostomy and subsequent Endo-SPONGE® (B. Braun Surgical S.A., Barcelona, Spain) vacuum assisted closure (EVAC) procedures [15]. All ileostomies were reversed within 1 year, without long term sequelae.

**Table 4:** An overview of the different fluorescent parameters for the overall group, and a comparison of fluorescent parameters in patients with and without anastomotic leakage

Fluorescence parameter	Total (*n* = 21)	No AL (*n* = 18)	AL (*n* = 3)*
Afferent loop			
Inflow parameters			
*t*_0_ (s)	25 (16–36)	26 (16–36)	38 (30–43)
* t*_max_ (s)	47 (35–62)	44 (34–65)	49 (38–58)
ttp (s)	17 (12–26)	16 (11–30)	20 (15–20)
* F*_max_ (AU)	56 (35–59)	51 (39–65)	32 (21–57)
Slope (AU/s)	2.4 (1.6–3.3)	2.4 (1.8–3.5)	1.4(0.8–2.5)
Outflow parameters			
t_90%_ (s)	7 (6–11)	6 (5–9)	10 (8–13)
t_80%_ (s)	12 (9–21)	10 (8–19)	20 (18–23)
Efferent loop			
Inflow parameters			
* t*_0_ (s)	27 (22–34)	29 (24–42)	33 (31–39)
* t*_max_ (s)	49 (36–63)	44 (36–65)	51 (43–57)
ttp (s)	17 (10–25)	17 (9–25)	22 (17–26)
* F*_max_ (AU)	40 (33–72)	39 (25–72)	47 (33–53)
Slope (AU/s)	1.9 (1.1–4.0)	1.6 (1.2–4.6)	2.2 (0.6–3.0)
Outflow parameters			
* t*_90%_ (s)	7 (5–13)	7(5–12)	8 (5–11)
* t*_80%_ (s)	12 (9–21)	12 (7–20)	15 (11–22)

*ttp* time-to-peak, *AU* arbitrary units, *AL* anastomotic leakage, *slope* mean slope

All numbers are medians and interquartile ranges between brackets. *signifies given median and range between brackets

Longer time values were observed in these few patients for both in- as out-flow parameters with respect to the afferent as efferent loop (see Table 4). The mean slope in the afferent loop was less steep in the AL group (1.4 versus 2.4 AU/s).

## Discussion

By quantifying ICG-FA of 21 patients undergoing IPAA surgery, we were able to determine quantitative in- and out-flow parameters within the pouch and explore the impact of lengthening measures requiring vascular ligation or previous inadvertent interruption of the ICA. In patients without vascular ligation of the ICA perfusion parameters between afferent and efferent loops were similar. However in patients with vascular ligation of the ICA both in- and out-flow time values were prolonged and the mean slope was less steep in the efferent loop of the pouch.

This study supports prior results, patients without vascular ligation show rapid in and outflow similarly in both afferent and efferent loop. In case of ligation, changes are observed predominantly in the efferent loop with in- and out-flow. This might be due to redirection of arterial flow through the arcade or potential venous obstruction. By contrast in case of AL, changes are also seen, but particularly in the afferent loop. ICG-FA is a promising tool to demonstrate adequate perfusion in gastrointestinal surgery [16]. This technique is potentially of added value during IPAA surgery, especially after vascular ligation [7, 8, 11, 17]. However, it remains challenging to differentiate between inflow (arterial) and outflow (venous) problems. Prior work indicates that it might be categorized in inflow problem (no ICG fluorescence), an outflow problem (delayed, but intact ICG flow), and adequate perfusion (rapid ICG flow) [7]. However, the use and interpretation of ICG-FA enhancement depends on subjective interpretation and in- and out-flow might affect each other.

This study shows that changes are visible in the shape of the curve and quantification parameters in the event of vascular ligation. Despite the fact that the curves in this study were not generated in real time, the clinical application for employing ICG-FA during IPAA might lie there. It can be challenging to determine intraoperatively whether the ICA is still intact. Particularly in patients with visceral obesity and in those cases where the arcade has been interrupted by ligation the descending branch of the ICA. In these patients ICG-FA can be applied to explore the integrity of the ICA and if needed to assess whether additional ligation of the interconnecting terminal ileal branches is possible. It is important to pay attention to keep the ICA intact when performing the colectomy since this might compromise perfusion and endanger the pouch's (specifically the efferent loop's) perfusion.

This is the first study to report on quantitative parameters of fluorescent time curves in IPAA surgery. All measurements were performed in a standardized manner and similar conditions. This produced valuable in and outflow ICG fluorescence data not reported before. The current study created new knowledge on pouch perfusion in general and explored the effect of lengthening measures on quantitative pouch perfusion patterns. The described technique is reproducible and will lead to a more objective interpretation of fluorescence angiography. A limitation of the study is that the cohort is too small to draw any firm conclusions regarding quantitative parameters and anastomotic leakage. However trends appear to be evident in the enhancement curve of the mainly the afferent loop of the pouch. If a quantitative threshold can be determined, this may select high risk patients for AL. Larger prospective trials should be carried out enabling multivariate analysis to identify a fluorescent threshold that may predict anastomotic leakage. This might influence clinical decision making intra- or post-operatively. For example changing a modified 2-stage into a 3-stage procedure or by taking pre-emptive measures postoperatively; for instance performing an early pouchoscopy for anastomotic inspection or preemptive endoSPONGE placement [11].

Besides, one fluorescence imaging system with standard settings was used. It is unknown how these findings relate to a setting with a different imaging system. In future studies, calibration of imaging systems is needed to identify differences in fluorescence read-out. Furthermore, in this study the fluorescent signal was still interpreted subjectively for intraoperative decisionmaking.

In conclusion, this study explored the effect of vascular ligation on pouch perfusion using quantitative ICG-FA. Evident changes in perfusion were found after ligation, mainly in the efferent loop of the pouch. ICG-FA can detect previous vascular ligation and might guide surgeons during clinical decision making intra- or post-operatively in IPAA.

## Supplementary Information

Below is the link to the electronic supplementary material.

Supplementary file1 (DOCX 13 kb)

## References

[1] Oresland T, Bemelman WA, Sampietro GM, Spinelli A, Windsor A, Ferrante M (2015). European evidence based consensus on surgery for ulcerative colitis. J Crohns Colitis.

[2] Fazio VW, Kiran RP, Remzi FH, Coffey JC, Heneghan HM, Kirat HT (2013). Ileal pouch anal anastomosis: analysis of outcome and quality of life in 3707 patients. Ann Surg.

[3] Sahami S, Buskens CJ, Fadok TY, Tanis PJ, de Buck van Overstraeten A, Wolthuis AM, et al (2016) Defunctioning ileostomy is not associated with reduced leakage in proctocolectomy and ileal pouch anastomosis surgeries for IBD. J Crohn's Colitis 10(7):779–785 10.1093/ecco-jcc/jjv201 26512136

[4] Reijntjes MA, Joosten JJ, Hompes R, Bemelman WA (2021). Additional lengthening measures and perfusion assessment during pouch surgery—a video vignette. Colorectal Dis.

[5] Shen R, Zhang Y, Wang T (2018). Indocyanine green fluorescence angiography and the incidence of anastomotic leak after colorectal resection for colorectal cancer: a meta-analysis. Dis Colon Rectum.

[6] Alekseev M, Rybakov E, Shelygin Y, Chernyshov S, Zarodnyuk I (2020). A study investigating the perfusion of colorectal anastomoses using fluorescence angiography: results of the FLAG randomized trial. Colorectal Dis.

[7] Slooter MD, van der Does de Willebois EML, Joosten JJ, Reijntjes MA, Buskens CJ, Tanis PJ (2022). Fluorescence perfusion assessment of vascular ligation during ileal pouch-anal anastomosis. Tech Coloproctol.

[8] Spinelli A, Carvello M, Kotze PG, Maroli A, Montroni I, Montorsi M (2019). Ileal pouch-anal anastomosis with fluorescence angiography: a case-matched study. Colorectal Dis.

[9] Hardy NP, Dalli J, Khan MF, Andrejevic P, Neary PM, Cahill RA (2021). Inter-user variation in the interpretation of near infrared perfusion imaging using indocyanine green in colorectal surgery. Surg Endosc.

[10] Lutken CD, Achiam MP, Osterkamp J, Svendsen MB, Nerup N (2021). Quantification of fluorescence angiography: toward a reliable intraoperative assessment of tissue perfusion—a narrative review. Langenbecks Arch Surg.

[11] Joosten JJ, Reijntjes MA, Slooter MD, Duijvestein M, Buskens CJ, Bemelman WA (2021). Fluorescence angiography after vascular ligation to make the ileo-anal pouch reach. Tech Coloproctol.

[12] Agha RA, Sohrabi C, Mathew G, Franchi T, Kerwan A, O'Neill N (2020). The PROCESS 2020 Guideline: Updating Consensus Preferred Reporting Of CasESeries in Surgery (PROCESS) Guidelines. Int J Surg.

[13] Zittan E, Wong-Chong N, Ma GW, McLeod RS, Silverberg MS, Cohen Z (2016). Modified two-stage ileal pouch-anal anastomosis results in lower rate of anastomotic leak compared with traditional two-stage surgery for ulcerative colitis. J Crohns Colitis.

[14] Elliott JT, Addante RR, Slobogean GP, Jiang SD, Henderson ER, Pogue BW (2020). Intraoperative fluorescence perfusion assessment should be corrected by a measured subject-specific arterial input function. J Biomed Opt.

[15] Gardenbroek TJ, Musters GD, Buskens CJ, Ponsioen CY, D'Haens GR, Dijkgraaf MG (2015). Early reconstruction of the leaking ileal pouch-anal anastomosis: a novel solution to an old problem. Colorectal Dis.

[16] Ris F, Liot E, Buchs NC, Kraus R, Ismael G, Belfontali V (2018). Multicentre phase II trial of near-infrared imaging in elective colorectal surgery. Br J Surg.

[17] Spinelli A, Cantore F, Kotze PG, David G, Sacchi M, Carvello M (2017). Fluorescence angiography during transanal trans-stomal proctectomy and ileal pouch anal anastomosis: a video vignette. Colorectal Dis.

