# Supplementary material for: Understanding fluorescence time curves during ileal pouch-anal anastomosis with or without vascular ligation
Source: Surg Endosc. 2023 Mar 14;37(7):5086–93. doi: 10.1007/s00464-023-09921-y (PMC10322949; doi:10.1007/s00464-023-09921-y)
Supplement: Supplementary file 1 — Supplementary file1 (DOCX 13 kb) [file 464_2023_9921_MOESM1_ESM.docx]

**Supplementary Table 1**. Fluorescence parameters of the patients with ligation of the interconnecting terminal ileal branches.

|  | **Pt 1** | **Pt 2** |
| --- | --- | --- |
| **Afferent loop** | | |
| Inflow parameters | | |
| t0 (sec) | 24 | 23 |
| t_max_ (sec) | 33 | 53 |
| ttp (sec) | 41 | 84 |
| F_max_ (AU) | 8.5 | 29 |
| Slope (AU/sec) | 2.8 | 2.3 |
| Outflow parameters | | |
| t_90%_ (sec) | 7 | 14 |
| t_80%_ (sec) | 13 | 23 |
| **Efferent loop** | | |
| Inflow parameters | | |
| t0 (sec) | 26 | 32 |
| t_max_ (sec) | 36 | 67 |
| ttp (sec) | 41 | 71 |
| F_max_ (AU) | 11 | 35 |
| Slope (AU/sec) | 2.3 | 1.5 |
| Outflow parameters | | |
| t_90%_ (sec) | 14 | 36 |
| t_80%_ (sec) | 36 | 71 |

Abbreviations: sec: seconds, ttp: time-to-peak, AU: arbitrary units, AL: anastomotic leakage, slope: mean slope
